# Supplementary material for: Extensive co-binding and rapid redistribution of NANOG and GATA6 during emergence of divergent lineages
Source: Nat Commun. 2022 Jul 23;13:4257. doi: 10.1038/s41467-022-31938-5 (PMC9308780; doi:10.1038/s41467-022-31938-5)
Supplement: Supplementary file 1 — Supplementary Information [file 41467_2022_31938_MOESM1_ESM.pdf]

Supplementary Information for Thompson et al. 2022

Supplementary Figure 1
Supplementary Figure 2
Supplementary Figure 3
Supplementary Figure 4
Supplementary Figure 5
Supplementary Figure 6
Supplementary Figure 7
Supplementary Figure 8

Supplementary Figure 1

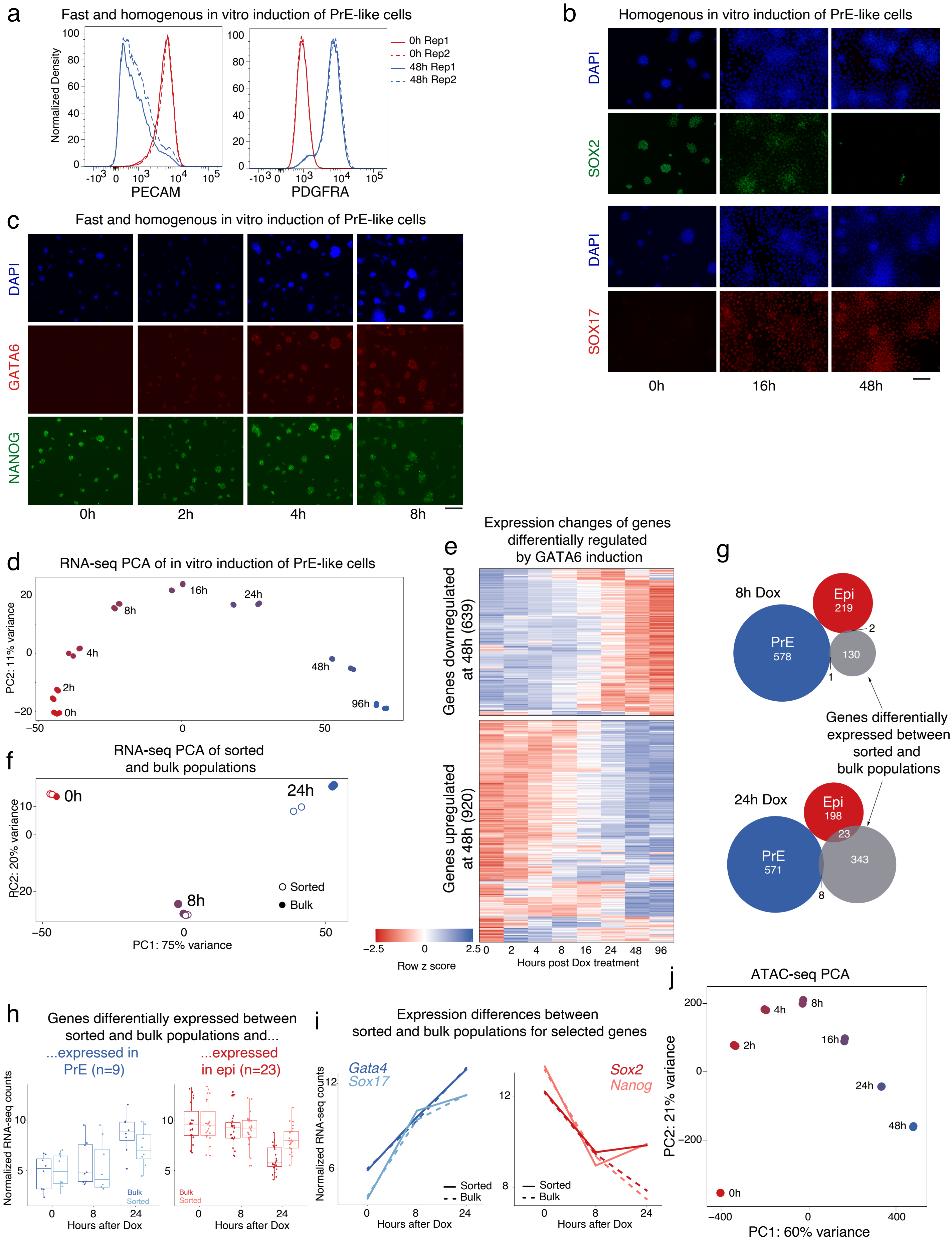

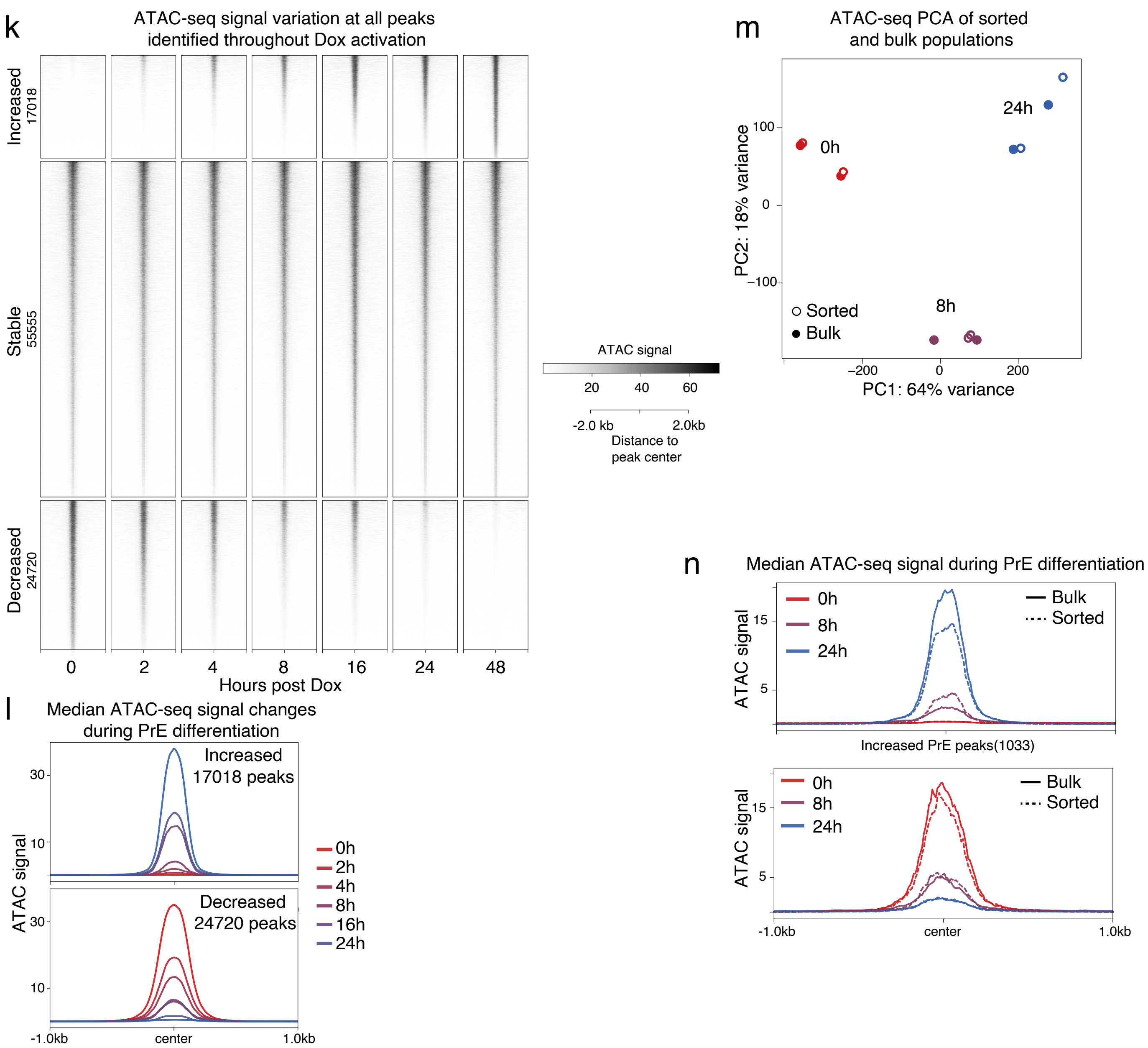

Supplementary Figure 1. GATA6-induction leads to quick and uniform transcriptional changes and remodelling of chromatin accessibility. a) Flow cytometry analysis at 0h and 48h on cells co-stained for the pluripotency surface marker, PECAM, and the PrE-specific surface marker, PDGFRA. Differentiation was associated with homogeneous loss of PECAM and gain of PDGFRA. **b)** SOX2 IF (green) showed that the Epi-state was quickly and homogeneously lost with concomitant gain in expression of the PrE marker, SOX17 (red). Representative images of experiment repeated three times. Scale bars in B and C represent 100μm. **c)** GATA6 (red) and NANOG (green) IF shows homogenous co-expression of the two TFs during early stages of differentiation. Representative image of experiment repeated three times. **d)** PCA of transcriptome changes accompanying differentiation as profiled by bulk RNA-seq shows that transcriptional changes were quickly initiated by 2h of GATA6 expression. **e)** Change in expression of all genes identified as differentially expressed during GATA6-induction represented as a heatmap of zscores for each gene. Plot in Fig.1c shows only the genes identified as differentially expressed between PrE and Epi in single cell blastocyst RNA-seq data. **f)** PCA plot comparing transcriptomes of unsorted bulk population (solid circles) and PDGFRA+ sorted cells at matched time points (open circles) shows that sorted cells were remarkably similar to cells assayed in bulk demonstrating the highly homogenous nature of GATA6-driven differentiation. **g)** Venn diagram comparing all genes that were differentially expressed in sorted cells compared to bulk population (grey) and the overlap of these genes with Epi (red) or PrE-specific genes (blue). Of all genes identified as differentially expressed in sorted and bulk populations, very few were part of the Epi (23) or PrE (9) gene network in vivo. **h)** Box plots comparing expression changes at the 9 PrE- and 23 Epi-specific genes that were identified as differentially expressed between bulk and sorted populations in e shows that even these genes still maintained similar direction of transcriptional changes during differentiation.Boxplots show minimum, maximum, median, first, and third quartiles. **i)** Line-plot showing that expression of key PrE (left) and Epi-specific (right) genes during differentiation in sorted and bulk populations at matched time points was very similar. **j)** PCA of chromatin accessibility throughout differentiation as assayed by ATAC-seq showing that just like the transcriptome, chromatin remodeling was quickly initiated by 2 hours of GATA6 expression. **k)** Heatmap showing the changes in ATAC-seq signal at all identified ATAC-seq peaks. Both accessibility gain and loss were initiated within two hours of GATA6-induced PrE differentiation. Changes in accessibility were categorized into chromatin regions that gained accessibility (increased), lost accessibility (decreased) and regions where accessibility remained unchanged when comparing 0h to 48h. **l)** Median ATAC-signal at each time-point plotted at the 17018 peaks across the genome that gained accessibility during differentiation (top panel) and at the 24720 peaks that lost accessibility. **m)** PCA of chromatin accessibility at matched time points from ATAC-seq performed in bulk (solid circles) and PDGFRA+ sorted (open circles) populations shows that in both cases chromatin was remodeled similarly. **n)** Plots comparing the median ATAC-seq signal from bulk and sorted populations at regions associated with PrE (top panel) and Epi genes (bottom panel). Peak regions are the same as in Fig 1e and this demonstrates that GATA6-induced cells homogenously reconfigured their chromatin.

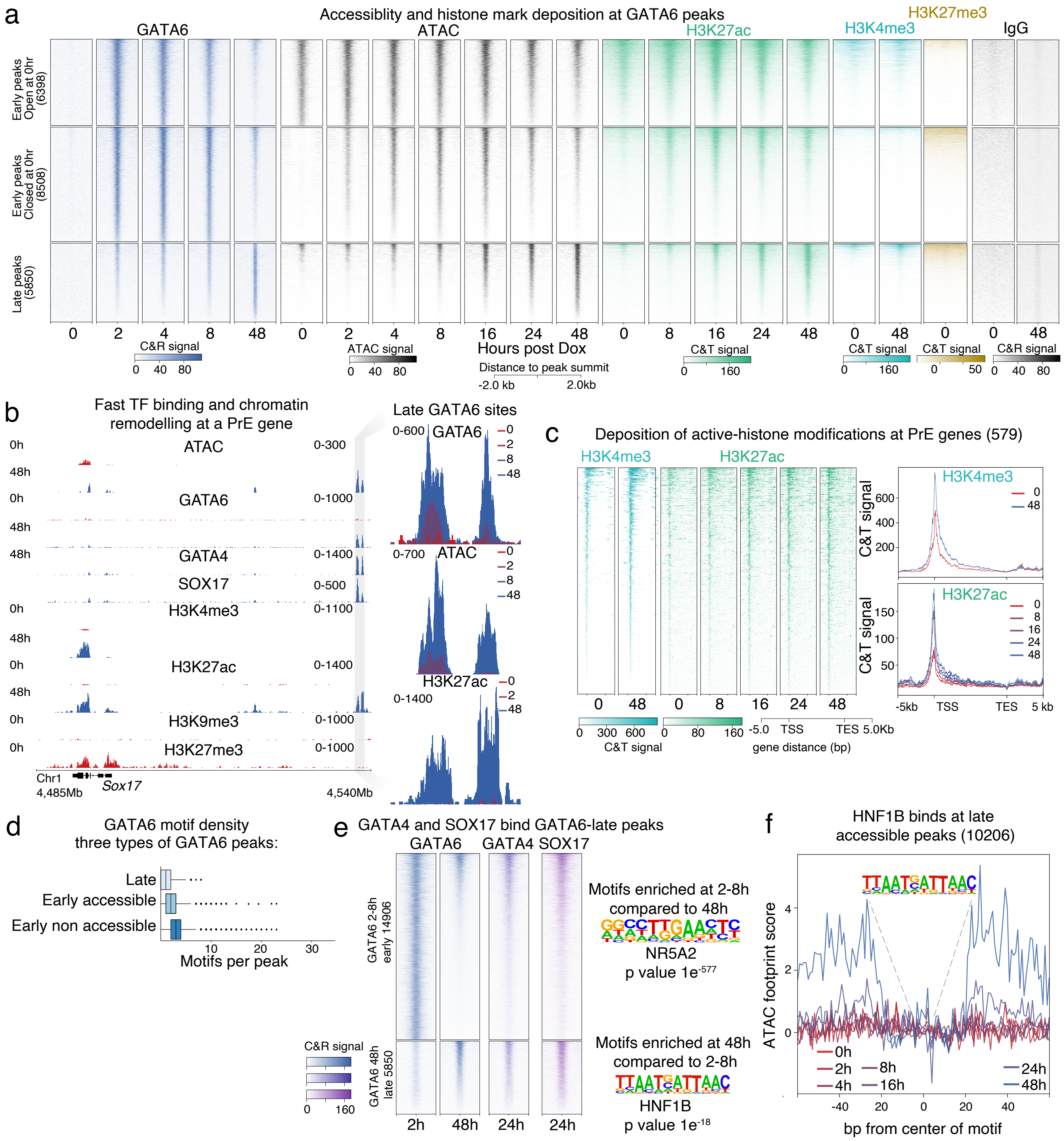

Supplementary Figure 2. GATA6 binding leads to gains in chromatin accessibility and H3K27ac deposition. a) Heatmap depicting changes in chromatin accessibility and distribution of histone marks at top 15000 GATA6 peaks identified across the mouse genome. GATA6 binding sites were classified into early peaks (bound by GATA6 within 8 hours of differentiation) and late peaks (bound by GATA6 only at 48 hours). Early peaks were further divided into those accessible before differentiation (Early peaks open at 0hr) and those that gained accessibility only after the induction of differentiation (early peaks closed at 0hr). **b)** Browser view at the *Sox17* locus showing the change in chromatin landscape at 48 hours of differentiation. Zoomed-in view of the grey highlighted region shows that this putative *Sox17* CRE is a late GATA6 binding site that is inaccessible at 0hrs and only reached high GATA6 binding and increase in chromatin accessibility by 48 hours. **c)** Heatmap showing deposition of H3K4me3 and H3H27ac at PrE gene-bodies as differentiation progressed. Median signal profile plots alongside show an increase of H3K4me3 and H3K27me3 at later time points in differentiation compared to the 0hr time-point. **d)** Density (number of motifs per peak) at three different GATA6 peak-types. Boxplots show minimum, maximum, median, first, and third quartiles. **e)** GATA4 and SOX17 bind most of GATA6 late-binding peaks. GATA4 and SOX17 24h CUT&RUN signal plotted at early (found between 2-8h) and late peaks (found only at 48h). GATA6 late peaks are enriched for the HNF1B motif when compared to motifs found from 2-8 hours using HOMER, while GATA6 early peaks are enriched in NR5A2 compared to late peaks. **f)** ATAC footprint score calculated using TOBIAS with the HNF1B motif at CREs that only became accessible at 48h. ATAC footprint score showing higher signal surrounding the HNF1B motif suggests HNF1B binding starting at 24h.

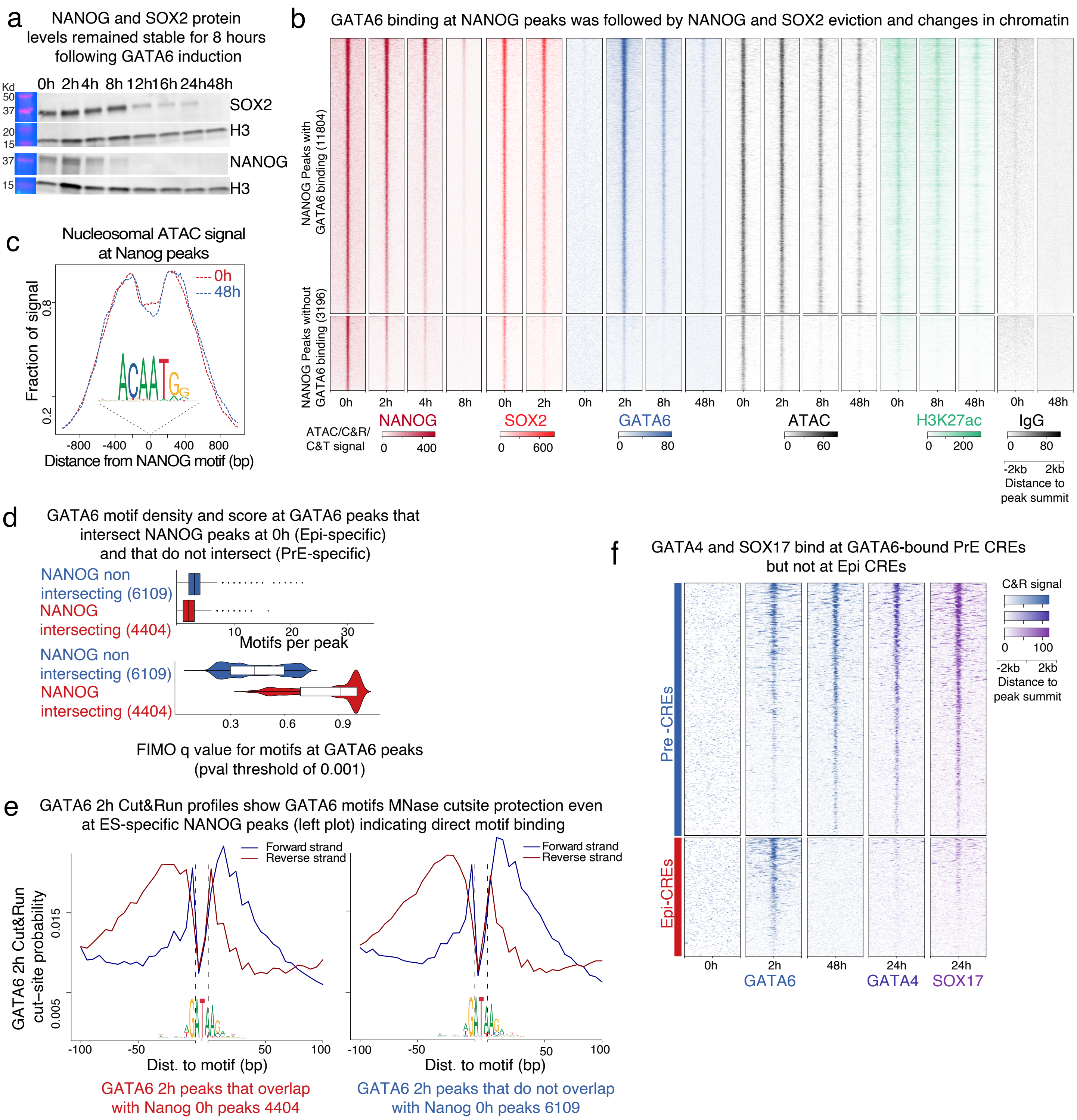

Supplementary Figure 3. GATA6 transiently binds most NANOG-bound regions prior to inactivation. a) Western blot of SOX2 and NANOG showing that protein levels of these two Epi-TFs is not decreased by 2h of differentiation and that NANOG is repressed faster than SOX2. Represenative image of experiment repeated two times. Uncropped blots can be found as source data in the provided souce data file file **b)** Heatmap depicting gradual loss of NANOG and SOX2 at NANOG peaks, concomitant with the transient binding of GATA6 at these loci, gradual loss of ATAC signal, and gradual depletion of H3K27ac with little change in H3K27me3 levels. The top 15000 NANOG peaks were divided into peaks where GATA6 overlapped with NANOG (top) and peaks where GATA6 did not overlap with NANOG. **c)** Nucleosomal fraction of ATAC-seq signal as determined by ATACseqQC at the indicated timepoints over NANOG motifs at NANOG-bound peaks shows minimal reshuffling of nucleosomes even at 48 hours post differentiation implying that NANOG motif occlusion by nucleosomes did not play a determinant role in silencing of the Epi transcriptional program. **d)** Density and motif strenght of GATA6 motifs at GATA6 peaks that NANOG did not bind at 0h (PrE-specific –blue) and peaks that were bound by NANOG at 0h (Epi-specific-red). Motif strength was calculated as a FIMO q value score that represents how close a motif is to the TF consensus motif. Epi-specific peaks also contained GATA6 motifs but at lower density and weaker strength compared to the GATA6 motifs at PrE-specific peaks. Boxplots show minimum, maximum, median, first, and third quartiles. **e)** CUT&RUN cutsite probability at GATA6 peaks that overlap with NANOG and are Epi CREs (left) and GATA6 motifs located in GATA6 peaks that did not overlap with NANOG and are PrE specific (right). In both cases, cutsite probability was similar and symmetric displaying protection of the GATA6 motif, suggesting that GATA6 motifs are indeed occupied by GATA6 in both types of peaks. **f)** GATA4 and SOX17 occupy PrE CREs but not Epi CREs suggesting they are not involved in repression of Epi CREs.

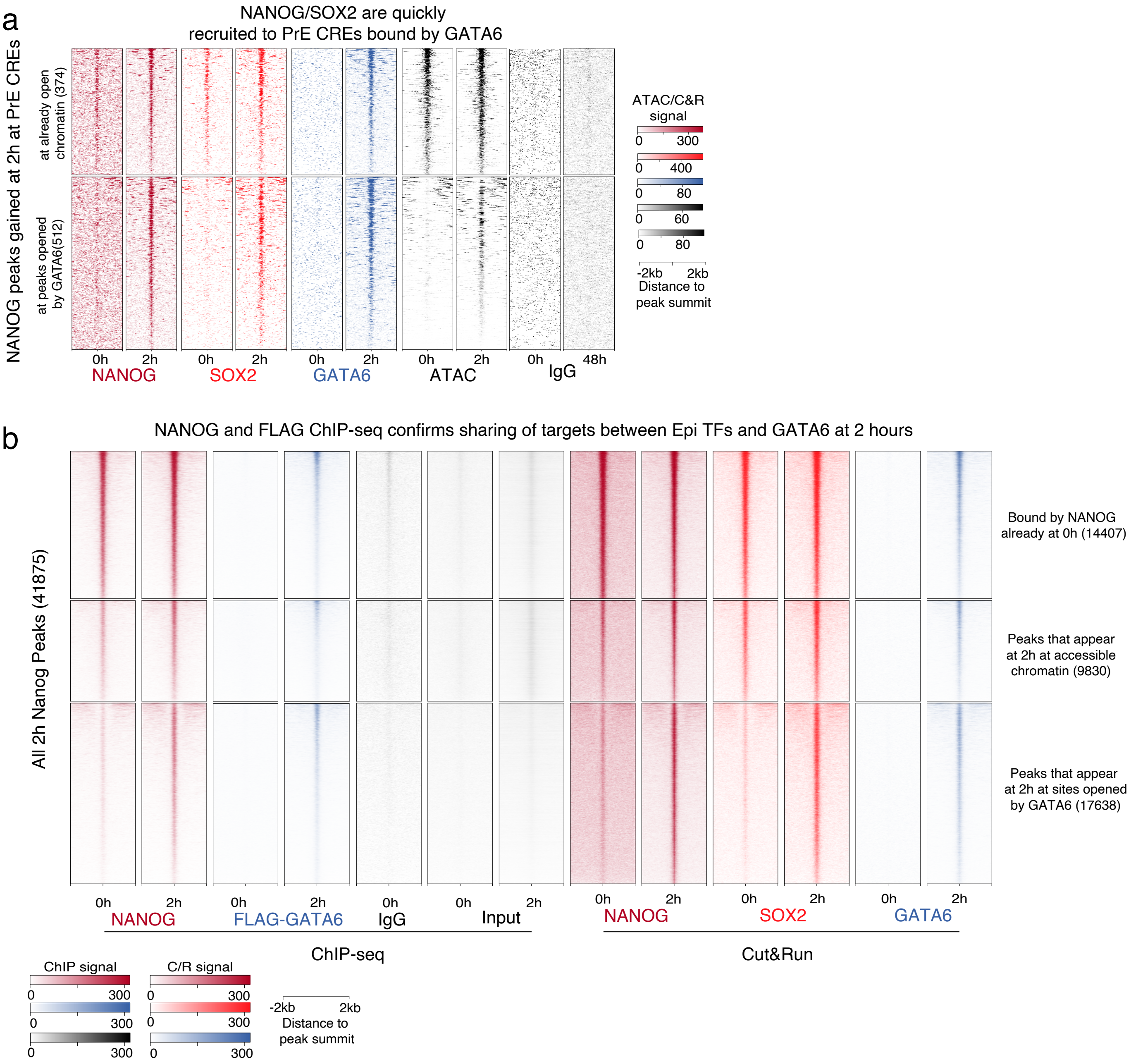

Supplementary Figure 4. Genome wide binding of NANOG and SOX2 to GATA6 targets. **a)** Heatmap showing de novo recruitment of NANOG and SOX2 at GATA6 CREs associated with PrE genes. **b)** Heatmap with same clusters as in Fig.4a showing that repositioning of NANOG to GATA6 peaks seen with CUT&RUN is also observed using ChIP-seq.

A

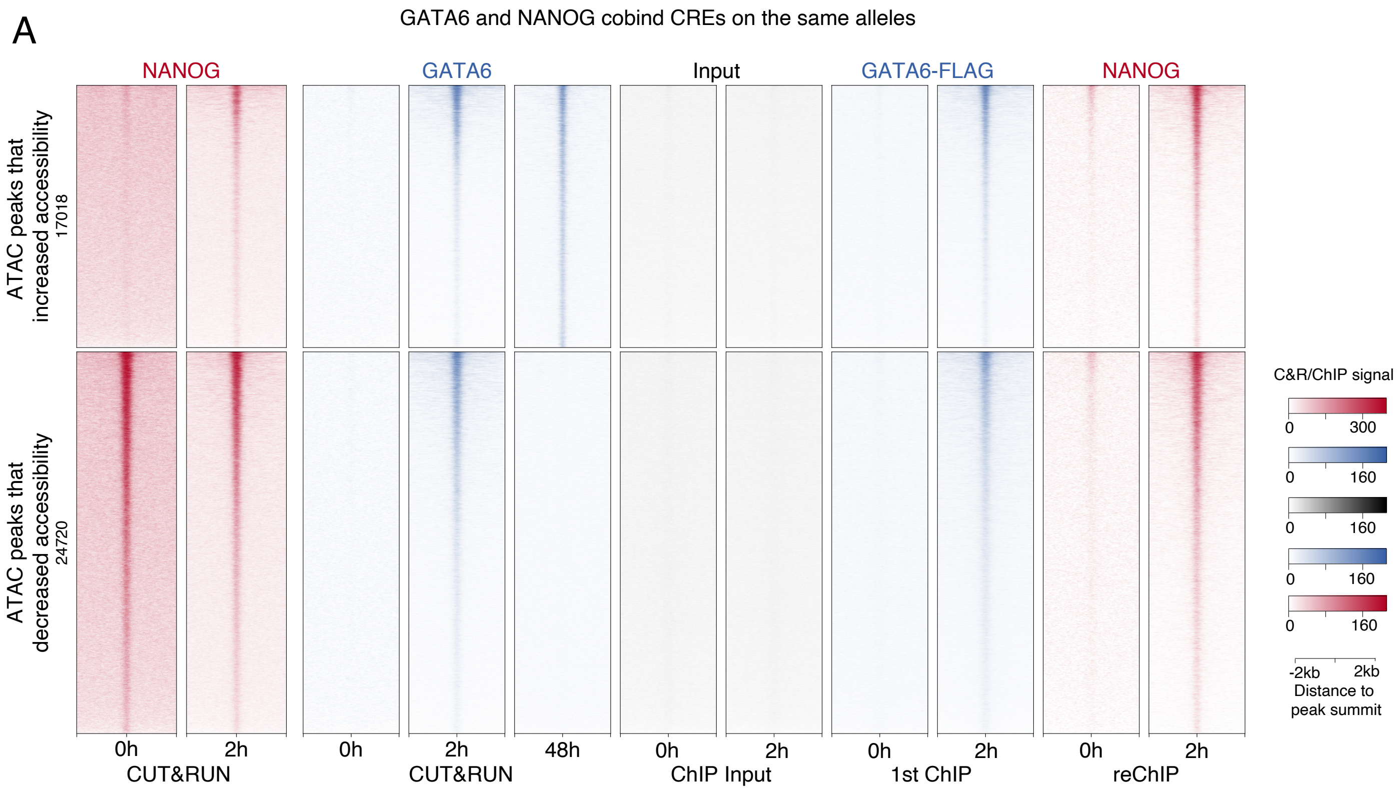

B

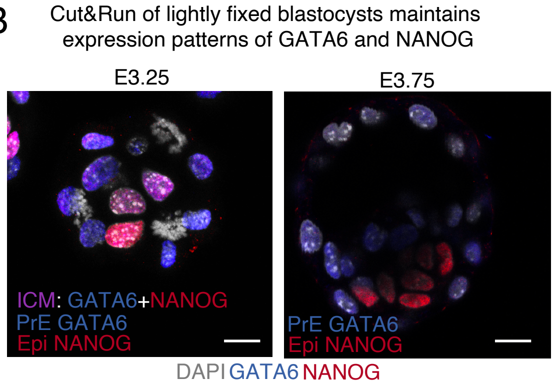

Supplementary Figure 5. Genome wide co-binding of NANOG and GATA6. **a)** Heatmap showing enrichment of FLAG-GATA6/NANOG reChIP signal at peaks identified genome-wide as ATAC-seq peaks that lost and gained accessibility during PrE differentiation. **b)** Example of blastocysts processed with our modified CUT&RUN protocol but hybridized with fluorescently-labeled secondary antibodies. Picture shows that light fixation maintained blastocyst structure and detected changes in tissue-specific expression of both GATA6 and NANOG. At E3.25, ICM cells that expressed both TFs can be seen (purple) as well as cells already committed to either TE (white), PrE (blue) or Epi(red). At later blastocyst stages, E3.5- E3.75 only TE, Epi and PrE-commited cells are found and no ICM cells are seen. Representative image of experiment repeated two times. Scale bar represents 20 μm

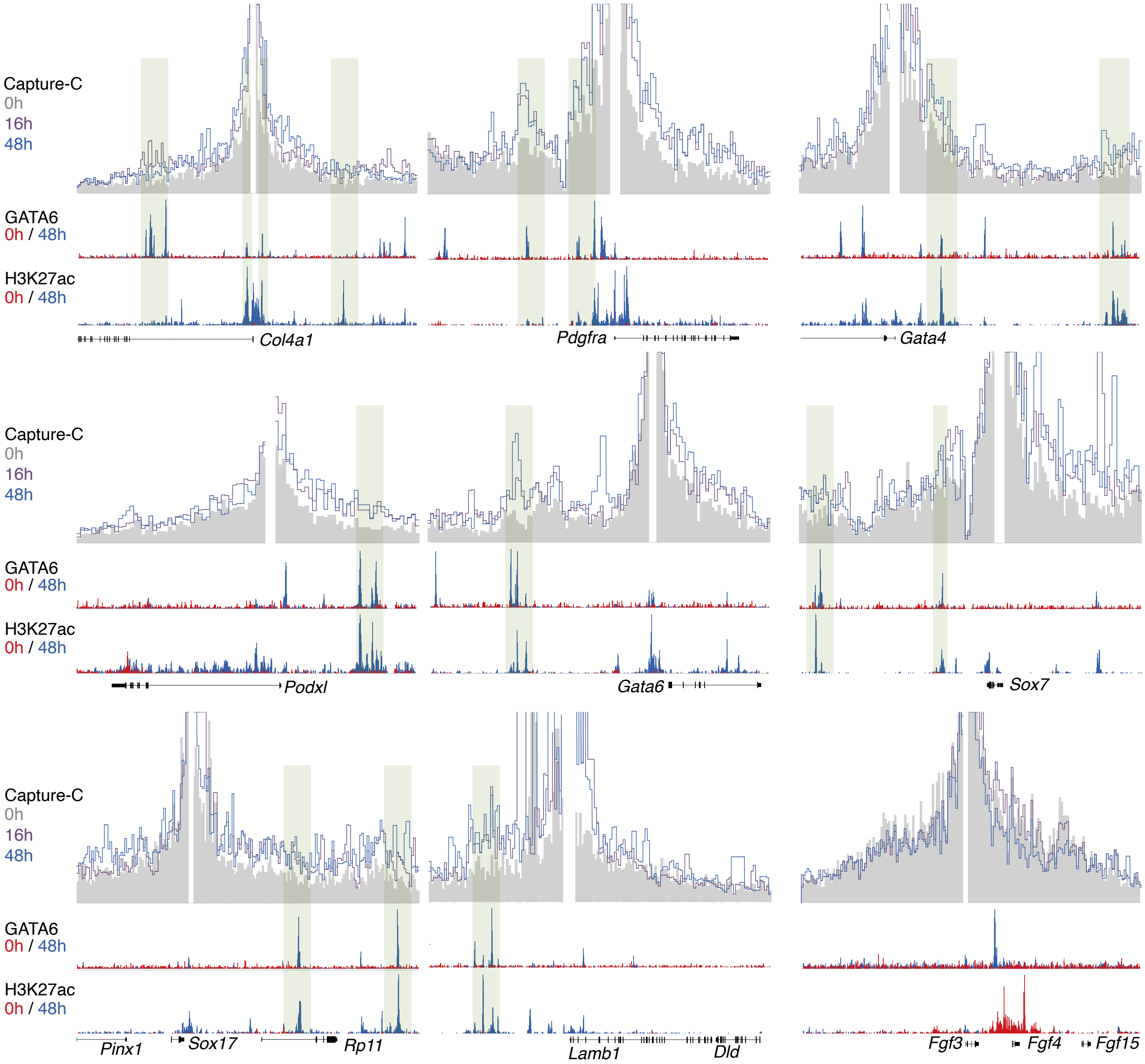

Supplementary Figure 6. PrE genes increased interaction frequency with GATA6-induced CREs within 16 hours of differentiation. All gene promoters increased interaction frequencies with regions that are bound by GATA6 and that gained H3K27ac during differentiation. The only exception is *Fgf3*, which is a PrE-specific gene but does not display distal CREs controlling its expression. Capture-C data is shown as the average signal of two replicates using 1kb bins. Green shaded area represents putative CREs with increased interactions with PrE-specific genes.

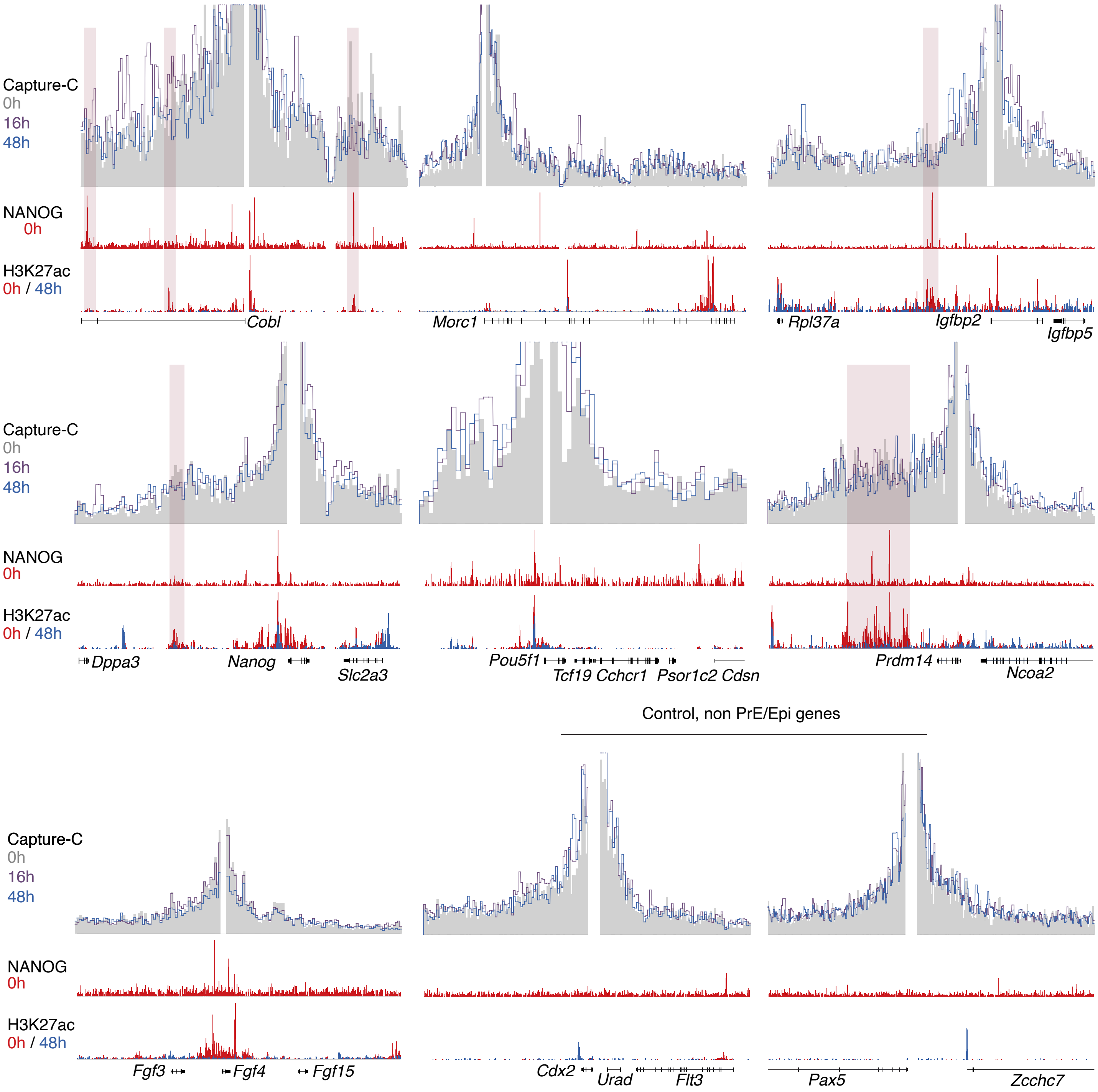

Supplementary Figure 7. Epi genes decreased interaction frequency with Epi-specific CREs within 16 hours of differentiation. Most Epi genes decreased interaction frequencies with regions that are bound by NANOG at 0h and that lose H3K27ac during differentiation. The exceptions were *Morc1*, *Fgf4* and *Pou5f1*. None of these genes show putative distal CREs possibly explaining higher stability of interactions. Two control genes that do not change expression during differentiation are shown and displayed no changes in interaction frequencies. Capture-C data is shown as the average signal of two replicates using bins of 1kb. Red shaded area represents putative CREs with decreased interaction frequency with Epi-specific genes.

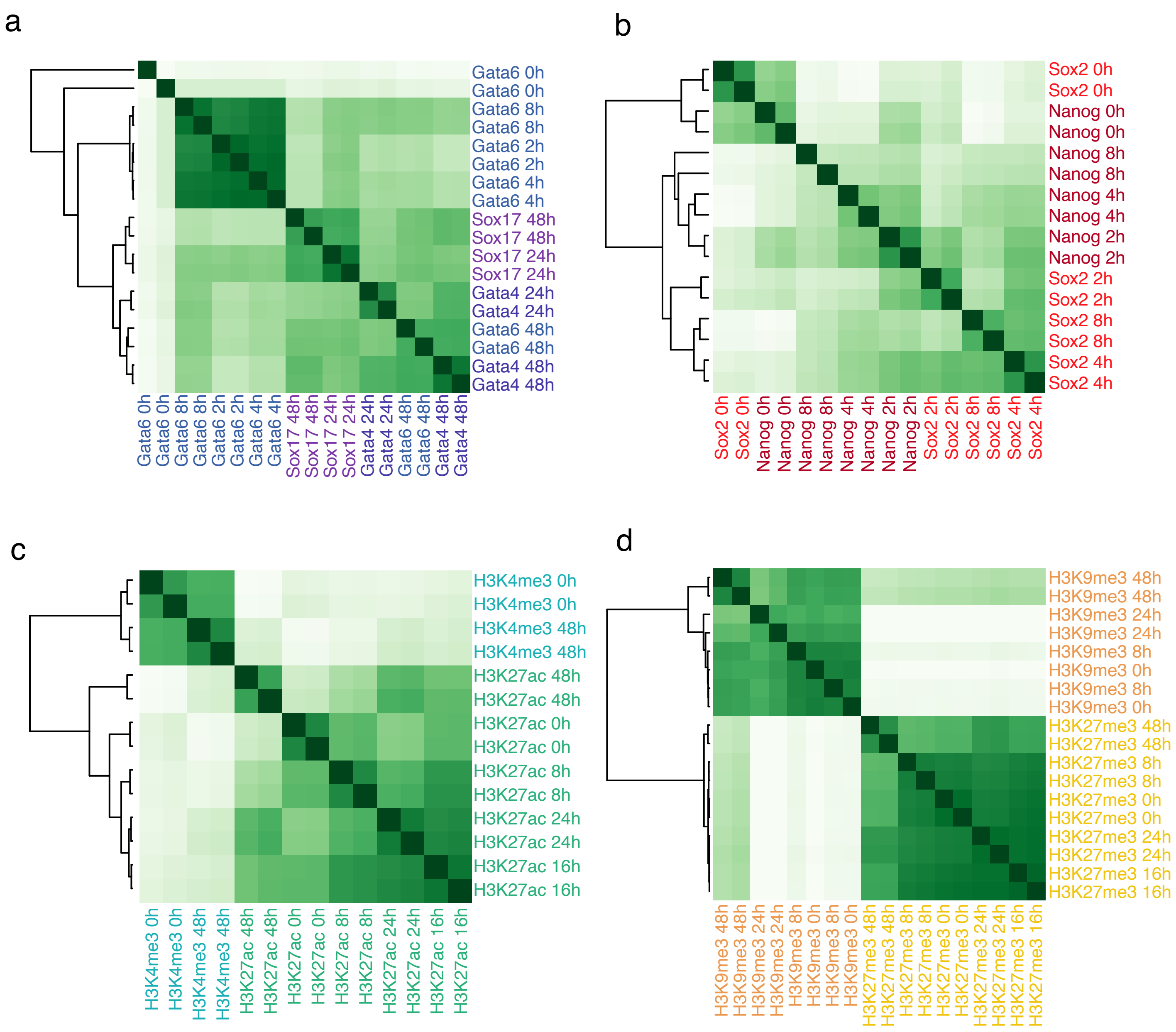

Supplementary Figure 8. Correlation analysis between replicates of CUT&RUN and CUT&TAG datasets. The R-package DiffBind was used to compare datasets. **a)** Correlation plot between the two replicates of each PrE TF profiled at the different time points. **b)** Correlation plot between the two replicates of each Epi TF profiled at the different time points. **c)** Correlation plot between the two replicates of active histone marks profiled by CUT&TAG at the indicated time points and **d)** Correlation plot between the two replicates of repressive histone marks profiled at the different time points.
