# Supplementary material for: Extensive co-binding and rapid redistribution of NANOG and GATA6 during emergence of divergent lineages
Source: Nat Commun. 2022 Jul 23;13:4257. doi: 10.1038/s41467-022-31938-5 (PMC9308780; doi:10.1038/s41467-022-31938-5)
Supplement: Supplementary file 3 — Description of Additional Supplementary Files [file 41467_2022_31938_MOESM3_ESM.pdf]

Description of Additional Supplementary Files

Supplementary Data1

a. Mapping information for each replicate used in the RNA-seq analysis.
b. List of genes identified as Epi or PrE specific in single-cell RNAseq datasets, and genes identifies as up- and down-regulated during in vitro PrE differentiation.
c. Mapping and peak information for each replicate on which ATAC-seq was performed.
d. Mapping and peak information for replicates from CUT&RUN analysis.
e. Mapping and peak information for replicates from CUT&TAG analysis.
f. Mapping and peak information for replicates from ChIP-seq analysis.
g. Mapping information for HiC and Capture-C analysis.
h. List of biotinylated oligos used in Capture-C
