# Supplementary material for: Extensive co-binding and rapid redistribution of NANOG and GATA6 during emergence of divergent lineages
Source: Nat Commun. 2022 Jul 23;13:4257. doi: 10.1038/s41467-022-31938-5 (PMC9308780; doi:10.1038/s41467-022-31938-5)
Supplement: Supplementary file 5 — Reporting Summary [file 41467_2022_31938_MOESM5_ESM.pdf]

nature portfolio

Corresponding author(s): Pedro P Rocha

Last updated by author(s): 6/2/2022

Reporting Summary

Nature Portfolio wishes to improve the reproducibility of the work that we publish. This form provides structure for consistency and transparency in reporting. For further information on Nature Portfolio policies, see our Editorial Policies and the Editorial Policy Checklist.

Statistics

For all statistical analyses, confirm that the following items are present in the figure legend, table legend, main text, or Methods section.

n/a	Confirmed	
☐	☒	The exact sample size (*n*) for each experimental group/condition, given as a discrete number and unit of measurement
☐	☒	A statement on whether measurements were taken from distinct samples or whether the same sample was measured repeatedly
☐	☒	The statistical test(s) used AND whether they are one- or two-sided<br>*Only common tests should be described solely by name; describe more complex techniques in the Methods section.*
☐	☒	A description of all covariates tested
☐	☒	A description of any assumptions or corrections, such as tests of normality and adjustment for multiple comparisons
☐	☒	A full description of the statistical parameters including central tendency (e.g. means) or other basic estimates (e.g. regression coefficient) AND variation (e.g. standard deviation) or associated estimates of uncertainty (e.g. confidence intervals)
☐	☒	For null hypothesis testing, the test statistic (e.g. *F*, *t*, *r*) with confidence intervals, effect sizes, degrees of freedom and *P* value noted<br>*Give P values as exact values whenever suitable.*
☒	☐	For Bayesian analysis, information on the choice of priors and Markov chain Monte Carlo settings
☐	☒	For hierarchical and complex designs, identification of the appropriate level for tests and full reporting of outcomes
☐	☒	Estimates of effect sizes (e.g. Cohen's *d*, Pearson's *r*), indicating how they were calculated

Our web collection on statistics for biologists contains articles on many of the points above.

Software and code

Policy information about availability of computer code

Data collection

The following software was used for data collection:
Microscopy: ZenBlue (2.6), Flowcytometry and Sorting: FACSdiva(v8.0.1),
Sequencing data was collected using the Illumina Hiseq2500 incorporated software.

Data analysis

Analysis for bulkRNAseq:
RNA-seq analysis including principal component analysis and identification of differentially expressed genes was performed using LCDB workflow (github.com/lcdb/lcdb-wf version 1.5.3).

Re-analysis of single-cell RNAseq from Nowotschin et al, 2019:
Data was downloaded as a cell x count matrix. The cell clustering determined in the study was downloaded and the count matrix was subset to only the relevant timepoints (embryonic day E3.5 and E4.5). For use in our study we renamed and combined some clusters based on cell identities as EPI, PrE or ICM: E3.5:1 = E3.5_ICM, E3.5:0 + E3.5:4 = E3.5_PrE, E3.5:2 = E3.5_EPI-E4.5:0 + E4.5:1 = E4.5_PrE, E4.5:2 = E4.5_EPI, E4.5:3 = E4.5_TE. Data was reanalyzed with the Seurat R package v3.1.2 using the standard workflow to find conserved markers and differentially expressed markers.

Analysis for ATAC-seq:
The ENCODE ATAC-seq pipeline (github.com/ENCODE-DCC/atac-seq-pipeline version 1.9.0) was used to process ATAC-seq.

Analysis for CUT&RUN, CUT&TAG, and ChIP-seq:
Paired-end 50bp reads were processed using Bowtie2 (2.4.5), with the following options (-N 1 --local --very-sensitive-local --no-unal --no-

mixed --no-discordant --phred33 -I 10 -X 700 -x). Reads that mapped to ENCODE mm10 blacklist regions were removed using Samtools (ver 1.15). Piccard ver 2.27.2 (broadinstitute.github.io/picard/) was then used to identify non-duplicated reads. Duplicate reads were removed for libraries generated from blastocysts but kept for all others. Diffbind (2.12.0) was used to analyze replicates by plotting pearson correlation of signal found over identified peaks. For generating bigwig files to visualize signal of transcription factors, DeepTools (3.5.1) was used to only retain fragments smaller than 120bp, while for histone modifications, fragments larger than 150bp were selected. MACS (2.1.1.20160309) was used to identify regions of enrichment using the narrow option for TFs and the broad option for histone modifications. Peak calling was done using CUT&RUN library generated using rabbit anti-rabbit antibody as control.

Analysis for HiC and CaptureC:
Data was processed using the Hi-Cpro (3.1.0) pipeline to produce a list of valid interactions pairs. This list was converted into cool and mcool files for visualization with higlass (1.11.7). Eigenvalues were calculated using homer (4.11.1) with 250kb bins and the runhicpca.pl script. For comparison between conditions, we used the gethiccorrDiff.pl script also at 250kb resolution.

Motif analysis:
All motif analysis was carried out using MEME (5.4.1).

Heatmaps:
Heatmaps for representing CUT&RUN, CUT&TAG, ChIP-seq and ATAC-seq were generated using Deeptools (3.5.1). Peak files for heatmaps were generated using bedtools (2.30.0). Coordinates for peak-regions of each heatmap are provided in source data.

For manuscripts utilizing custom algorithms or software that are central to the research but not yet described in published literature, software must be made available to editors and reviewers. We strongly encourage code deposition in a community repository (e.g. GitHub). See the Nature Portfolio guidelines for submitting code & software for further information.

Data

Policy information about availability of data

All manuscripts must include a data availability statement. This statement should provide the following information, where applicable:

- Accession codes, unique identifiers, or web links for publicly available datasets
- A description of any restrictions on data availability
- For clinical datasets or third party data, please ensure that the statement adheres to our policy

The datasets generated in this study can be accessed on the Gene Expression Omnibus database under the accession number GSE181104 (https://www.ncbi.nlm.nih.gov/geo/query/acc.cgi?acc=GSE181104). Source data are provided with this paper.

Mouse Vista enhancers were downloaded from the VISTA database (https://enhancer.lbl.gov/) and ENCODE CREs were obtained from the UCSC genome browser.

Human research participants

Policy information about studies involving human research participants and Sex and Gender in Research.

Reporting on sex and gender

Use the terms sex (biological attribute) and gender (shaped by social and cultural circumstances) carefully in order to avoid confusing both terms. Indicate if findings apply to only one sex or gender; describe whether sex and gender were considered in study design whether sex and/or gender was determined based on self-reporting or assigned and methods used. Provide in the source data disaggregated sex and gender data where this information has been collected, and consent has been obtained for sharing of individual-level data; provide overall numbers in this Reporting Summary. Please state if this information has not been collected. Report sex- and gender-based analyses where performed, justify reasons for lack of sex- and gender-based analysis.

Population characteristics

Describe the covariate-relevant population characteristics of the human research participants (e.g. age, genotypic information, past and current diagnosis and treatment categories). If you filled out the behavioural & social sciences study design questions and have nothing to add here, write "See above."

Recruitment

Describe how participants were recruited. Outline any potential self-selection bias or other biases that may be present and how these are likely to impact results.

Ethics oversight

Identify the organization(s) that approved the study protocol.

Note that full information on the approval of the study protocol must also be provided in the manuscript.

Field-specific reporting

Please select the one below that is the best fit for your research. If you are not sure, read the appropriate sections before making your selection.

☒ Life sciences ☐ Behavioural & social sciences ☐ Ecological, evolutionary & environmental sciences

For a reference copy of the document with all sections, see nature.com/documents/nr-reporting-summary-flat.pdf

Life sciences study design

All studies must disclose on these points even when the disclosure is negative.

Sample size	The number of replicates used in each assay and statistics used in analysis of the different datasets are described under the corresponding subsections in methods. Similarity between replicates is detailed in SupplementaryData1 and depicted in FigS7. No statistical method was used to predetermine sample size. We performed 4 replicates of RNA-seq which showed very little variability between replicates. As this method is more sensitive to variation than our other techniques we decided that duplicates would suffice and indeed we always observed very high reproducibility.
Data exclusions	No data were excluded from the analyses except for samples where QC failed because of low signal or high background i.e. low FRIP (fraction of reads that fall into a peak). In those cases the experiment was repeated.
Replication	Experiments on replicates was performed independently and numbers can be found in the methods. All replications were sucessfull and no data had to be excluded for not replicating previous findings.
Randomization	Randomization was not necessary for our study as it does not involve a case-control cohort experiment.
Blinding	Because our study did not involve targeted deletion of any factor, in cells or in mice, blinding was not necessary. All data was analysis in an unbiased fashion.

Reporting for specific materials, systems and methods

We require information from authors about some types of materials, experimental systems and methods used in many studies. Here, indicate whether each material, system or method listed is relevant to your study. If you are not sure if a list item applies to your research, read the appropriate section before selecting a response.

Materials & experimental systems

n/a	Involved in the study	
☐	☒	Antibodies
☐	☒	Eukaryotic cell lines
☒	☐	Palaeontology and archaeology
☐	☒	Animals and other organisms
☒	☐	Clinical data
☒	☐	Dual use research of concern

Methods

n/a	Involved in the study	
☐	☒	ChIP-seq
☐	☒	Flow cytometry
☒	☐	MRI-based neuroimaging

Antibodies

Antibodies used

Antibodies used for CUT&RUN (at dilution 1:50):
GATA6 (R&D Systems, AF1700), NANOG (Active Motif, 61419), SOX2 (Millipore, ab5603), OCT4 (abcam, ab19857), GATA4 (Santa Cruz Biotech, sc25310), SOX17 (R&D Systems, AF1924), H3K9me3 (Abcam, ab8898)

Antibodies used for CUT&TAG (at dilution 1:50):
H3K4me3 (Active Motif, 39159), H3K27ac (Abcam, ab4729), H3K27me3 (Cell Signaling, 9733T)

Antibodies used for ChIP-seq (2 micrograms):
FLAG-M2 (Sigma, F3165-2MG), NANOG (Active Motif, 61419)

Antibodies used for immunofluorescence (Primary antibodies diluted 1:500; secondary antibodies diluted 1:1000):
SOX17 (R&D Systems, AF1924), SOX2 (Millipore, ab5603), GATA6 (R&D Systems, AF1700), Donkey-anti-rabbit-AF488 (Invitrogen, A21206), Donkey-anti-goat-AF555 (Invitrogen, A21432)

Antibodies used for Western blot (Primary antibodies diluted 1:1000; secondary antibodies diluted 1:5000):
Rabbit anti-NANOG (Abcam, ab80892), Rabbit anti-SOX2 (abcam, ab92494), histone-H3 (abcam, ab176842), Anti-Rabbit-HRP (Cell signaling technologies, 7074P2)

Antibodies used for flow-cytometry (at dilution 1:1000):
PDGFRA-FITC (ThermoFisher Scientific, 11-1401-82) and PECAM-APC (BD Pharmigen, clone MEC 13.3, 551262)

Validation

As described in (https://www.nature.com/articles/s41467-019-09982-5), all antibodies used in this study were commercially available, have been verified by the manufacturer by Western blotting or by peptide ELISA described and used in several studies as mentioned on the manufacturer's specification sheets.

Eukaryotic cell lines

Policy information about cell lines and Sex and Gender in Research

Cell line source(s)	KH2 ES cells engineered to contain Doxycycline-inducible GATA6 were a gift from Kathy Niakan (The Francis Crick Institute, UK)
Authentication	mES cells were phenotypically characterized
Mycoplasma contamination	No mycoplasma testing was performed
Commonly misidentified lines (See ICLAC register)	This cell line is not listed in the ICLAC Database of Cross-contaminated or Misidentified Cell Lines.

Animals and other research organisms

Policy information about studies involving animals; ARRIVE guidelines recommended for reporting animal research, and Sex and Gender in Research

Laboratory animals	Blastocysts were collected from 4-5 weeks old C57BL/6N females six days after PMSG/HCG injections, mated with 8-10 week old males.
Wild animals	No wild animals were used in this study
Reporting on sex	As we need to pool embryos before it is possible to genotype/differentiate genders data generated here was analyzed not considering sex as a variable
Field-collected samples	No field collected samples were used in the study.
Ethics oversight	All mouse studies were performed according to NIH and PHS guidelines and only after protocols were approved by the Animal Care and Use Committees of the National Cancer Institute and Eunice Kennedy Shriver National Institute of Child Health and Human Development.

Note that full information on the approval of the study protocol must also be provided in the manuscript.

ChIP-seq

Data deposition

- [x] Confirm that both raw and final processed data have been deposited in a public database such as GEO.
- [x] Confirm that you have deposited or provided access to graph files (e.g. BED files) for the called peaks.

Data access links
May remain private before publication.

https://www.ncbi.nlm.nih.gov/geo/query/acc.cgi?acc=GSE181104

Files in database submission

The following files correspond to ChIP-seq experiments:

GSM5484529 JT_499_0h_ixen_Nanog_ChIP_merged_rep1
GSM5484530 JT_500_0h_ixen_Nanog_ChIP_merged_rep2
GSM5484531 JT_505_0h_ixen_IgG_ChIP_merged_rep1
GSM5484532 JT_506_0h_ixen_merged_Input
GSM5484533 JT_507_2h_ixen_Nanog_ChIP_merged_rep1
GSM5484534 JT_508_2h_ixen_Nanog_ChIP_merged_rep2
GSM5484535 JT_511_2h_ixen_merged_Input
GSM5819708 JT_677_gata6RFPline1_0h_1%input_rechip
GSM5819709 JT_678_gata6RFPline1_2h_1%input_rechip
GSM5819710 JT_679_gata6RFPline1_0h_FLAG_chip1_rep1
GSM5819711 JT_680_gata6RFPline1_0h_FLAG_chip1_rep2
GSM5819712 JT_681_gata6RFPline1_2h_FLAG_chip1_rep1
GSM5819713 JT_682_gata6RFPline1_2h_FLAG_chip1_rep2
GSM5819714 JT_683_gata6RFPline1_0h_FLAG_chip1_Nanog_chip2_rep1
GSM5819715 JT_684_gata6RFPline1_0h_FLAG_chip1_Nanog_chip2_rep2
GSM5819716 JT_685_gata6RFPline1_2h_FLAG_chip1_Nanog_chip2_rep1
GSM5819717 JT_686_gata6RFPline1_2h_FLAG_chip1_Nanog_chip2_rep2

The following files correspond to CUT&RUN experiments:

GSM5484568 Gata6_0h_ixen_CR_DL_12_rep1
GSM5484569 Gata6_0h_ixen_CR_JT_247_rep2

GSM5484570 Gata6_2h_ixen_CR_DL_73_rep1
GSM5484571 Gata6_2h_ixen_CR_DL_74_rep2
GSM5484572 Gata6_4h_ixen_CR_DL_75_rep1
GSM5484573 Gata6_4h_ixen_CR_DL_76_rep2
GSM5484574 Gata6_8h_ixen_CR_DL_77_rep1
GSM5484575 Gata6_8h_ixen_CR_DL_78_rep2
GSM5484576 Gata6_48h_ixen_CR_JT_137_rep1_merged
GSM5484577 Gata6_48h_ixen_CR_JT_138_rep2_merged
GSM5484578 JT_333_Gata4_ixen_24hr_rep1
GSM5484579 JT_334_Gata4_ixen_24hr_rep2
GSM5484580 DL_248_Sox17_24h_rep1
GSM5484581 DL_249_Sox17_24h_rep2
GSM5484582 DL_44_CR_Nanog_0h_rep1
GSM5484583 DL_45_CR_Nanog_0h_rep2
GSM5484584 DL_46_CR_Nanog_2h_rep1
GSM5484585 DL_47_CR_Nanog_2h_rep2
GSM5484586 DL_48_CR_Nanog_4h_rep1
GSM5484587 DL_49_CR_Nanog_4h_rep2
GSM5484588 DL_50_CR_Nanog_8h_rep1
GSM5484589 DL_51_CR_Nanog_8h_rep2
GSM5484590 DL_53_CR_Sox2_0hrs_rep1
GSM5484591 DL_54_CR_Sox2_0hrs_rep2
GSM5484592 DL_55_CR_Sox2_2hrs_rep1
GSM5484593 DL_56_CR_Sox2_2hrs_rep2
GSM5484594 DL_57_CR_Sox2_4hrs_rep1
GSM5484595 DL_58_CR_Sox2_4hrs_rep2
GSM5484596 DL_59_CR_Sox2_8hrs_rep1
GSM5484597 DL_60_CR_Sox2_8hrs_rep2
GSM5484598 JT_141_CR_IgG
GSM5484599 JT_495_Bl6SpOv_scE3.5Blastocysts120x_CR_0_1perPFA_Gata6
GSM5484600 JT_517_Bl6SpOv_scE3.5Blastocysts160x_CR_0_1perPFA_Gata6
GSM5484564 DL_17_CR_H3K9me3_0h_rep1
GSM5484565 JT252_CR_H3K9me3_0h_rep2
GSM5484566 JT_133_CR_H3K9me3_48hr_rep1
GSM5484567 JT_134_CR_H3K9me3_48hr_rep2
GSM5819706 JT_671_Bl6SpOv_E3.5Blastocysts130x_CR_0.1perPFA_Nanog
GSM5819707 JT_672_Bl6SpOv_E4.5Blastocysts55x_CR_0.1perPFA_Gata6

The following files correspond to CUT&TAG experiments:

GSM5484546 DL_212b_CT_H3K4me3_0h_rep1
GSM5484547 DL_213b_CT_H3K4me3_0h_rep2
GSM5484548 DL_232_CT_H3K4me3_48h_rep1
GSM5484549 DL_233_CT_H3K4me3_48h_rep2
GSM5484550 DL_216b_CT_H3K27me3_0h_rep1
GSM5484551 DL_217_CT_H3K27me3_0h_rep2
GSM5484552 DL_236_CT_H3K27me3_48h_rep1
GSM5484553 DL_237_CT_H3K27me3_48h_rep2
GSM5484554 DL_214b_CT_H3K27ac_0h_rep1
GSM5484555 DL_215b_CT_H3K27ac_0h_rep2
GSM5484556 DL_220_CT_H3K27ac_8h_rep1
GSM5484557 DL_221_CT_H3K27ac_8h_rep2
GSM5484558 DL_224_CT_H3K27ac_16h_rep1
GSM5484559 DL_225_CT_H3K27ac_16h_rep2
GSM5484560 DL_228_CT_H3K27ac_24h_rep1
GSM5484561 DL_229_CT_H3K27ac_24h_rep2
GSM5484562 DL_234_CT_H3K27ac_48h_rep1
GSM5484563 DL_235_CT_H3K27ac_48h_rep2

Genome browser session (e.g. UCSC)	no longer applicable

Methodology

Replicates	For all datasets generated from mES cells, at least two replicates were performed

Sequencing depth

CUT&RUN and CUT&TAG samples were sequenced at 10-15 million reads per samples (paired-end 50). ChIP-seq samples were sequenced at ~25 million reads per samples (paired-end 50). Reads per sample are listed in SupplementaryData1

Antibodies

Antibodies used for CUT&RUN (at dilution 1:50):
GATA6 (R&D Systems, AF1700), NANOG (Active Motif, 61419), SOX2 (Millipore, ab5603), OCT4 (abcam, ab19857), GATA4 (Santa Cruz Biotech, sc25310), SOX17 (R&D Systems, AF1924), H3K9me3 (Abcam, ab8898)

Antibodies used for CUT&TAG (at dilution 1:50):
H3K4me3 (Active Motif, 39159), H3K27ac (Abcam, ab4729), H3K27me3 (Cell Signaling, 9733T)

Antibodies used for ChIP-seq (2 micrograms):
FLAG-M2 (Sigma, F3165-2MG), NANOG (Active Motif, 61419)

Peak calling parameters

Described in Methods

Data quality

Described in Methods with details listed in SupplementaryData1

Software

Paired-end 50bp reads were processed using Bowtie2 (2.4.5), with the following options (-N 1 --local --very-sensitive-local --no-unal --no-mixed --no-discordant --phred33 -I 10 -X 700 -x). Reads that mapped to ENCODE mm10 blacklist regions were removed using Samtools (ver 1.15). Piccard ver 2.27.2 (broadinstitute.github.io/picard/) was then used to identify non-duplicated reads. Duplicate reads were removed for libraries generated from blastocysts but kept for all others. Diffbind (2.12.0) was used to analyze replicates by plotting pearson correlation of signal found over identified peaks. For generating bigwig files to visualize signal of transcription factors, DeepTools (3.5.1) was used to only retain fragments smaller than 120bp, while for histone modifications, fragments larger than 150bp were selected. MACS (2.1.1.20160309) was used to identify regions of enrichment using the narrow option for TFs and the broad option for histone modifications. Peak calling was done using CUT&RUN library generated using rabbit anti-rabbit antibody as control.

Flow Cytometry

Plots

Confirm that:

- [x] The axis labels state the marker and fluorochrome used (e.g. CD4-FITC).
- [x] The axis scales are clearly visible. Include numbers along axes only for bottom left plot of group (a 'group' is an analysis of identical markers).
- [ ] All plots are contour plots with outliers or pseudocolor plots.
- [ ] A numerical value for number of cells or percentage (with statistics) is provided.

Methodology

Sample preparation

Cells were harvested using 10mM EDTA made in PBS. After incubation in EDTA for 10 mins, cells were dislodged by pipetting to ensure a suspension of single cells, following which cells were spun and resuspended in 500µl of MACS buffer (PBS, 2% FBS, 1mM EDTA). Cells were double stained with preconjugated PDGFRA-FITC (ThermoFisher Scientific, 11-1401-82) and PECAM-APC (BD Pharmigen, clone MEC 13.3, 551262) antibodies by adding 0.5µl of each antibody. Cells were incubated at 40C for 20 mins following which they were washed twice in MACS buffer. Cell pellets were finally resuspended in 800µl MACS buffer and analyzed for proportions of FITC positive/APC negative cells.

Instrument

BD FACSAria

Software

FlowJo

Cell population abundance

An small portion of the sorted cells were re-analyzed by Flow Cytometry again in the BD FACSAria to confirm a purity of over 95%

Gating strategy

Procedure for gating to sort dsRed2 positive cells: FSC/SSC -> Singlets -> Live cells (DAPI negative) -> gating on dsRed2-high. Un-induced cells (no dsRed2 expression) was used as negative control.

- [ ] Tick this box to confirm that a figure exemplifying the gating strategy is provided in the Supplementary Information.
